# Developing the Observatory Test of Capacity, Performance, and Developmental Disregard (OTCPDD) for Children with Cerebral Palsy

**DOI:** 10.1371/journal.pone.0151798

**Published:** 2016-03-24

**Authors:** Kuan-Chun Liu, Hao-Ling Chen, Tien-Ni Wang, Jeng-Yi Shieh

**Affiliations:** 1 School of Occupational Therapy, College of Medicine, National Taiwan University, Taipei, Taiwan; 2 Department of Physical Medicine and Rehabilitation, National Taiwan University Hospital, Taipei, Taiwan; University Children's Hospital Tuebingen, GERMANY

## Abstract

**Purpose:**

The purpose of this study was to develop a reliable and valid instrument, named the Observatory Test of Capacity, Performance, and Developmental Disregard (OTCPDD), for measuring the amount and quality of use of affected upper limb functions in the daily routines of children with CP.

**Methods:**

Forty-eight participants (24 children with CP and 24 matched typically developing children) were recruited. The OTCPDD was administered twice (the spontaneous use condition first, followed by the forced use condition) on children with CP. Their parents were asked to complete the Pediatric Motor Activity Log-Revised (PMAL-R). The internal consistency, the intrarater and interrater reliabilities, and the convergent and discriminate validities were measured.

**Results:**

The internal consistency (Cronbach’s alpha) and the intrarater and interrater reliabilities were higher than 0.9 for all of the OTCPDD scores. The convergent validity was confirmed by significant correlations between the OTCPDD and the PMAL-R. For the discriminant validity, significant differences (p<0.05) were found between children with CP and typically developing children.

**Conclusions:**

The results support that the OTCPDD is a reliable and valid observation-based assessment. The OTCPDD, which uses bimanual daily living activities, is able to represent the children’s general affected hand functions (including capacity, performance, and developmental disregard) in their daily routines.

## Introduction

Cerebral palsy (CP), defined broadly as “a non-progressive motor impairment syndrome caused by a problem in the developing brain,” is the most common pediatric physical disability, with an incidence of 2.11 per 1,000 live births [1]. Children with hemiparesis or substantially greater deficit in one upper extremity than the other have been reported to account for a considerable 15.3~36% of the CP population [2–5]. Such children tend not to use the affected hand, which leads to a decrease in strength and motor control and further interferes with the development of bilateral coordination in multiple domains (e.g., play, self-care, and daily activities) [6]. The tendency of a child to underuse or disuse the affected upper limb is called developmental disregard [7–9].

Developmental disregard has been referred to as a process wherein a child learns not to use the affected hand during the development of motor skills [2, 9, 10]. It is observed in 50%-90% in children with hemiplegic cerebral palsy [8, 11]. Children with hemiplegia have received positive reinforcement from successful experiences of using the unaffected hand and negative reinforcement from unsuccessful experiences of using the affected hand. The long-term interaction between positive and negative reinforcements causes them to develop adaptive strategies of preferring only to use the unaffected hand in daily activities, regardless of the preserved capacity of the affected hand [12]. This lack of spontaneous use of the affected hand in bimanual tasks is known as developmental disregard [8, 13, 14], and the discrepancy between the ability to use (forced-use capacity) and actual use (real-world performance) of the affected hand is identified as the severity of the developmental disregard.

Few assessments have been developed to investigate the phenomenon of developmental disregard in children with CP. The Pediatric Motor Activity Log-Revised (PMAL-R) [15], revised from the Motor Activity Log used in adult stroke patients [16, 17], is a questionnaire-based instrument for assessing how much and how well a child actually uses his or her more-affected arm in daily life. While the PMAL-R gathers meaningful data on the spontaneous use of the affected upper limb in daily life, it also includes many unilateral tasks (e.g., turning on or off a light, picking up small objects, and pointing at a target). Since it is common to use the dominant hand to perform unilateral tasks, even in the normal population, evaluating the use of the affected hand with bimanual activities could more directly capture the real upper limb performance [11, 14, 18]. Furthermore, the PMAL-R, a questionnaire-based test, does not measure the motor capacity of a child’s affected upper limb. Thus, the discrepancy between capacity and performance cannot be obtained using this instrument.

The revised Video-Observation Aarts and Aarts module: Determine Developmental Disregard (the VOAA-DDD-R) [8, 11, 18], an observation-based instrument, has also been developed to measure upper limb capacity, performance, and developmental disregard in children with CP. The VOAA-DDD-R includes two tasks: a bead-stringing task and a muffin-decorating task. The bead-stringing task, which requires children to use both upper limbs together to string beads, can be used to evaluate the capacity of the affected upper limb. The muffin-decorating task, which is designed to encourage (stimulating, but not requiring) children to use both upper limbs together, can be used to evaluate spontaneous use of the affected upper limb. Developmental disregard is calculated by the discrepancy between the capacity (the bead task) and the performance (the muffin task) [8].

Although the VOAA-DDD-R is able to measure the capacity, performance, and developmental disregard of affected limbs in children with CP, a major concern is that it lacks daily living tasks that would reflect the children’s actual upper limb functions in daily routines. In addition, since task properties could influence a child’s motor performance [19, 20], the use of two different tasks to estimate the discrepancy between capacity and performance might lead to construct errors. In order to reduce the influence of task properties, having participants perform the same task in both the spontaneous use and the forced use conditions might be a more feasible and reasonable way to determine the phenomenon of developmental disregard.

In summary, the current promising assessments for measuring developmental disregard can be classified as subjective and objective tools. PMAL-R, the subjective questionnaire, obtains data on parental perceptions of how much and how well a child actually uses his or her more-affected arm by daily tasks. However, it includes many unilateral tasks that may not capture the real upper limb performance of the affected hand [11, 14, 18]. The VOAA-DDD-R, the objective observational-based assessment, examines the capacity, performance, and developmental disregard of the affected upper limb in children with CP. However, it does not include enough activities of daily living to represent the use of the affected upper limb in daily routines.

Thus, the purposes of the current study were to develop a reliable and valid assessment, named the Observatory Test of Capacity, Performance, and Developmental Disregard (OTCPDD), for measuring the performance of affected upper limbs in the daily routines in children with CP. To be specific, the OTCPDD retained the important features of the PMAL-R [15] in terms of measuring the amount and quality of the use of the affected hand functions in daily activities, but we have modified all items into bimanual activities. In addition, the OTCPDD used the same scoring structure as the VOAA-DDD-R in terms of capacity, performance, and developmental disregard [8] and employed an observation-based manner, but we have expanded the number of items from 2 to 18 to sufficiently represent the use of the affected upper limb in daily routines.

The psychometric properties of the OTCPDD, including the internal consistency, the intrarater and interrater reliabilities, and the convergent and discriminate validities, were investigated. In addition, since the subjective questionnaire might reflect participants’ (or their caregivers’) real perceptions of their motor performance in daily life and might provide important information for evidence-based rehabilitation [21, 22], we also developed an OTCPDD item-matched self-report questionnaire, named the Questionnaire of Developmental Disregard (QDD), to provide supplemental subjective information. The results of the OTCPDD and the QDD were also compared.

## Methods

### Participants

Forty eight children (24 children with hemiplegic CP and 24 typically developing children) were recruited for the current study. The inclusion criteria for children with CP were (1) diagnosis of hemiplegic CP and (2) age between 5 and 13 years old. Children were excluded if they could not understand or execute the tasks because of intellectual disability. In addition, 24 age-matched typically developing children were recruited to establish the norm reference. Informed consent was obtained in writing from guardians of the children who participated in this study. All procedures in this study were approved by the Institutional Review Board of National Taiwan University Hospital.

### Testing tasks of the OTCPDD

The OTCPDD was designed to investigate the capacity, performance, and developmental disregard through observation-based evaluations of children with hemiplegic CP performing daily bimanual tasks. The testing tasks were selected according to two principles. First, the tasks should be common in or related to the daily living activities of school-aged children. In this case, the results could be more closely related to daily performance. Three main types of children’s daily activities were identified: academic-related, self-care related, and play-related. Second, the selected tasks should be bimanual activities. For this principle, the development and component of bimanual movement was considered and reviewed [23]. In addition, the object characteristics (e.g., size, weight, texture, shape, and location) were also considered because they can influence the way a participant manipulates objects. Thus, the selected objects were chosen according to these characteristics and were tested on the typically developing children first to ensure their feasibility. After several rounds of pilot testing and modification of the chosen activities, 18 tasks were finally selected (S1 Appendix).

### Administration of the OTCPDD

The setup for the OTCPDD included a camera, a chair, and a table. The camera was placed ipsilateral to the affected or non-dominant side of the participants, at 45° from the midline and a height of 1 meter, to capture the movements of the affected upper limb. An adjustable table and chair were provided for the participant. The height of the table was approximately equal to that of the participant’s chest to allow free use of the upper limbs, and the chair was set to a height at which the participant’s feet could rest firmly on the floor. Standardized instructions for each item were provided. The participants were then asked to perform each task naturally (real-world performance) first, followed by a second trial (forced-use capacity) in which the child was guided to use both hands if necessary.

### Scoring of the OTCPDD and QDD

The scoring of the OTCPDD included (1) the amount and quality of use of the affected hand in the spontaneous condition for measuring performance (P-AOU, P-QOM); (2) the amount and quality of the use of the affected hand in the forced-use condition for measuring capacity (C-AOU, C-QOM); and (3) the discrepancy between the amount and quality of use of the affected hand in real-world performance and the forced-use capacity to represent the severity of the developmental disregard (DD-AOU, DD-QOM). To be specific, the DD-AOU was calculated as the C-AOU score minus the P-AOU score, and the DD-QOM was calculated as the C-QOM score minus the P-QOM score.

For the amount of use (AOU), raters viewed the video recording and scored the occurrences of 10 functional motor components (reach, grasp, hold & carry, release, stabilize, adjust, catch/throw, manipulate, press, and pinch). In addition, these 10 functional motor components were further classified into the two categories including 5 basic functions: reach, grasp, hold & carry, release, and stabilize; and 5 advanced functions: adjust, catch/throw, manipulate, press, and pinch. The ten functional motor components were scored on a binary scale (0/1). If the particular motor component was observed at least one time, the item was scored as 1 point, and if the component was not observed, it was scored as 0 points. Considering that each item might have its own motor components, two raters jointly reviewed 15 videos of typically developing children while performing the OTCPDD. The two raters scored and discussed all ten of the motor components across the 18 tasks and identified the main components of each item. The maximum scores of each item ranged from 5 to 9 points, which meant that each item had 5 to 9 main motor components (S2 Appendix).”

Quality of movement (QOM) was scored on a six-point scale (0–5) to record the general quality of the participants’ movement strategies for each item. The criteria were set in terms of motor coordination, motor accuracy, muscle tone, the role of the affected upper limb, compensatory strategies, and associated movements. Five points implied that the performance was equal to that of a typically developing child, and 0 points indicated that no movements or only associated movements of the affected hand were observed (S1 Appendix). To be specific, a score of 5 points indicated that no abnormal pattern was observed and that the both upper limbs could play complementary roles of working hands for the intended purposes in an accurate and coordinated fashion. A score of 4 points indicated that both upper limbs could play the roles of working hands for the intended purposes, but the movement was slightly slow and uncoordinated, possibly with atypical motor patterns. A score of 3 points indicated that the affected upper limb could play the role of assisting hand during the whole process or that the performance was significantly clumsy or slow due to the influence of abnormal muscle tone or atypical patterns. A score of 2 points meant that the participant finished the task with external compensatory strategies, including using other body parts or external support to assist the affected upper limb, or that the affected upper limb could only partially play the role of an assisting hand. A score of 1 point meant that the participant tried to use the affected upper limb but failed to achieve functional use, and a score of 0 points indicated that no movements or only associated movements were observed.

In addition, the scores of the QDD are generally based on the scoring design of the PMAL-R, which includes the AOU and QOM subscales. These two subscales are scored on an 11-point scale (0–5, the minimum point unit being 0.5). A score of 0 represents nonuse or non-functional quality, and a score of 5 represents an AOU or QOM equivalent to that of a typically developing child (S3 Appendix).

### Reliability

The internal and external consistencies of the scoring system were examined in this study. The internal consistency of the scoring system was examined with Cronbach’s alpha to see if the selected items could consistently reflect the planned construct. The external consistency of the scoring system was examined by comparing the intra-rater and inter-rater reliabilities with the intraclass correlation coefficient (ICC). The intra-rater reliability was examined by calculating the agreement between scores of the same video rated twice within two weeks by the same rater. The inter-rater reliability was examined by calculating the agreement between scores of the same video by two raters.

### Validity

The convergent validity was examined by calculating the Spearman rank correlation coefficient between the OTCPDD and the PMAL-R [15]. A higher correlation between these two tools indicated comparable abilities to measure the use of the affected hand in daily activities. The discriminant validity was determined by comparing the scores of children with CP with those of typically developing children by Mann-Whitney U test.

### Procedure

For children with hemiplegic CP, the OTCPDD was administered twice. The first session comprised administration of all items in the spontaneous condition for measuring performance, followed by the forced-use condition for measuring capacity. The children’s parents were asked to complete the PMAL-R and DDQ. For typically developing children, only one session of the OTCPDD was administered. The duration of the evaluation period was 20 to 40 minutes for children with CP and 15 minutes for typically developing children.

## Results

### Participants

In all, 48 participants, 24 children with hemiplegic CP and 24 matched typically developing children, were recruited for the study. Their characteristics are shown in Table 1. No significant differences were found between these two groups (p>0.05).

**Table 1:** Demographic data of the children with cerebral palsy (CP) and the typically developing children (TDC).

	CP (n = 24)	TDC (n = 24)	*p*
**Age (months)**			
Mean (SD)	108.68(25.33)	106.15 (17.40)	0.550^a^
Range	63.7~155.9	80.7~139.7	
**Gender, n**			
Male	13	11	0.564^b^
Female	11	13	
**Affected Side, n**			
Right	11	-	-
Left	13	-	-
**Dominant Side, n**			
Right	-	22	-
Left	-	2	-
**MACS, n**			
I	3	-	-
II	11	-	-
III	10	-	-
**OTCPDD**			
Performance AOU	76.29(28.12)	109.46 (4.19)	<0.001^a^
Performance QOM	58.21(22.88)	99.13 (1.94)	<0.001^a^
Capacity AOU	82.08(21.10)	-	-
Capacity QOM	62.79(18.03)	-	-
DD AOU	5.79(7.91)		
DD QOM	4.75(6.26)		

SD = standard deviation

MACS = manual ability classification system

OTCPDD = Observatory Test of Capacity, Performance, and Developmental Disregard

AOU = amount of use of the affected upper limb

QOM = quality of movement of the affected upper limb

DD = Developmental Disregard

^a^Mann-Whitney U test

^b^chi-square test

### Reliability

The internal consistency (the Cronbach’s alpha) of the OTCPDD was higher than 0.9 on all of the OTCPDD scores, indicating excellent internal consistency in each measure (Table 2). The intrarater and interrater reliabilities were also excellent, with ICCs ranging from 0.902 to 0.995 (Table 2).

**Table 2:** Reliabilities of the Observatory Test of Capacity, Performance, and Developmental Disregard (OTCPDD).

OTCPDD Scores	Internal Consistency	Reliabilities	
	Cronbach’s alpha	Intrarater ICC (95% CI)	Interrater ICC (95% CI)
Performance AOU	0.955	0.995(0.989~0.998)	0.989(0.974~0.995)
Performance QOM	0.981	0.992(0.981~0.996)	0.981(0.957~0.992)
Capacity AOU	0.935	0.995(0.989~0.998)	0.986(0.967~0.994)
Capacity QOM	0.983	0.987(0.971~0.995)	0.971(0.932~0.987)
DD-AOU	-	0.973(0.938~0.988)	0.922(0.819~0.966)
DD-QOM	-	0.968(0.927~0.986)	0.902(0.773~0.958)

ICC = Intraclass correlation coefficient

CI = confidence interval

DD = Developmental Disregard

AOU = amount of use of the affected upper limb

QOM = quality of movement of the affected upper limb

### Validity

The convergent validity results demonstrated moderate correlations between the OTCPDD and the PMAL-R (r = 0.487, p<0.05 for AOU and r = 0.446, p<0.05 for QOM). The discriminant validity results were supported by the significant differences in the OTCPDD AOU and QOM between children with hemiplegic CP and typically developing children (Table 1). These findings demonstrate the ability of the instrument to discriminate between the two groups.

### The Item-Matched Subjective Questionnaire: DDQ

High correlations were found between the DDQ and the PMAL-R (r = 0.716, p<0.05 for AOU and r = 0.813, p<0.05 for QOM). With regard to the relationship between objective and subjective assessments, significant moderate to high correlations were found between the OTCPDD AOU and DDQ AOU (r = 0.508, p<0.05) and between the OTCPDD QOM and DDQ QOM (r = 0.513, p<0.05).

## Discussion

The results of this study indicate that the OTCPDD is a reliable and valid assessment for evaluating the capacity, performance, and developmental disregard in children with CP. The unique feature of the OTCPDD is that it detects the general affected hand functions by assessing the performance of bimanual daily-relevant tasks (including learning, self-care, and play activities) in children with CP. Developmental disregard is defined as a tendency to underuse or disuse the affected upper limb in daily routines, which is not parallel to preserved capacity in daily life [7, 8, 13, 14]. Thus, the OTCPDD, combining the strengths of the PMAL-R [15] (using daily activities) and the VOAA-DDD-R [8] (using observation-based bimanual activities), was developed to provide comprehensive investigation of developmental disregard.

The high Cronbach’s alpha (alpha>0.9) indicated that the 18 items reflected coherent abilities. The external consistency of the scoring system was supported by the excellent intra-rater and inter-rater reliabilities of all of the OTCPDD scores (ICCs>0.9) found in our study. The excellent external consistency indicates that the OTCPDD is able to consistently reflect subjects’ performance regardless of external factors, such as the scoring performance of the same rater across a period of time or the scoring performances of different raters. These results were contributed by our preliminary work on the OTCPDD scoring system. In that work, two raters together reviewed 15 videos of the OTCPDD and scored the motor components and movement qualities for all of the 18 tasks thoroughly. After reaching consensus on the scoring criteria, a new rater, who was blind to the study purpose, was trained for the investigation of the inter-rater reliability. The high inter-rater reliability (between the OTCPDD developer and a new rater) not only confirmed the stability of the OTCPDD scoring but also supported the feasibility of rater training.

The moderate significant correlation (r = 0.446–0.487, p<0.05) between the OTCPDD and the PMAL-R supports the consistency of these two tests. This moderate correlation coefficient meets our expectation that the OTCPDD is able to assess how much a child actually uses his or her more-affected arm in daily activities when the testing items (unilateral or bilateral) and methods (subjective or objective) are different. Further studies investigating the convergent validities between the OTCPDD and other assessments should be conducted to verify this finding.

As for the discriminant validity, a significant difference (p<0.05) was found between typically developing children and children with CP in the scores of the OTCPDD AOU and OTCPDD QOM. This finding supports the ability of the OTCPDD to discriminate between children in these two groups. In addition, we further analyzed the discriminant ability of each of the ten motor components, and our findings echoed those of Aart et al.’s [15, 16] studies, which found that even fundamental motor functions (e.g., grasp, hold, and release) were sensitive enough to discriminate the motor performances of typically developing children from those of children with CP. However, advanced motor functions (e.g., adjust, manipulate, and pinch) might be necessary for documentation of changes or improvements in motor functions over time. Further studies will be necessary to examine that possibility.

The OTCPDD is the first assessment to estimate the real discrepancy between capacity and performance on the same tasks. We asked a child to perform the tasks under natural conditions to measure real-world performance, followed by a second trial, in which the child was guided to use both hands to measure forced-use capacity. This approach could reduce internal errors and decrease the influence of task properties [19]. The discrepancy between the use of the affected hand in real-world performance and the forced-use capacity is represented by the scores of the DD-AOU and DD-QOM. The DD-AOU score indicates the quantity of the preserved capacities that children with CP disregard. For example, if a child obtains a DD-AOU score of 10 points, it indicates that this child with CP disregards 10 motor components in real-world performance. This child has the capacity for the 10 hidden motor components but does not transfer that capacity to real-world performance. In addition, the score of the DD-QOM indicates the quality of movement for the above preserved capacities. These scores could help researchers and therapists further to understand the discrepancy between motor capacity and motor performance of children and to evaluate the potential for improvement of the affected upper limbs. The results may also guide clinicians to provide adequate rehabilitation programs.

Interestingly, we also found that the scores of the DD-AOU were negatively associated with those of the P-QOM (r = -0.855, p<0.05), indicating that the poorer the quality of movement of the children, the more preserved capacities they may disregard. This finding may be explained by the suggestion of Houwink et al. [7] that children with poorer movement quality may experience over-loading of cognitive efforts while performing a task. Children with hemiplegic CP have relatively few experiences using their affected hand that make them prefer not to use their affected upper limb when the demand of task is high. Otherwise, they have to put their efforts both on their affected hand as well as the demanding of task at the same time. In this case, the automatizing of motor capacity into motor performance was limited. These unique scores could further help researchers and clinicians to understand children’s movement quality and motor capacity and present the potential capability for stimulation of the use of the affected limb in their daily routines. The results may also provide clinical proof that intensive training of the affected limb (such as Constraint-Induced Movement Treatment) can help to improve the movement quality and thus reduce the phenomenon of developmental disregard [24, 25].

This study also investigated the feasibility of the matched questionnaire (DDQ). The significant high correlations between the DDQ and the PMAL-R (r = 0.716–0.813, p<0.05) support the promising subjective outcomes of the DDQ in measuring affected hand functions in daily routines. In addition, the moderate to high relationships between the subjective and the objective outcomes of the same item design, the OTCPDD and the DDQ, (r = 0.508–0.513, p<0.05) fit our expectations and are in line with findings based on typically developing children [22]. This study also adds further support to the idea that parents’ perceptions of their children’s motor skill performance are correlated moderately-to-largely with their children’s real performance, not only in typically developing children but also in children with CP. Furthermore, according to our observations, we found that all the inconsistencies occurred in those parents who had children with moderate movement quality. Such children tend to have fundamental functional skills but perform tasks more slowly or clumsily. Thus, their parents tended to provide relatively lower scores on the DDQ, even when the child performed well in observed tests (the OTCPDD). The results might be explained by the high expectations of the parents and their magnification of the children’s motor deficits in reference to typically developing children. Further studies should investigate the potential factors (e.g., parental education, expectations, and stress level) that lead to this inconsistency to verify this hypothesis.

A few limitations of this study warrant consideration. First, the participants were a convenience sample, and only children with mild-to-moderate CP were included, which might limit the generalizability of the findings. A larger sample of children with CP with differing levels of motor impairment is necessary to verify the results of the current study. Second, the OTCPDD was developed mainly to measure a child’s motor functions based on daily relevant activities with minimum cognitive demands. However, data on cognitive function (e.g., IQ) were not collected in this study. Since cognition may be an important factor in children’s development, future studies should investigate the influence of cognitive levels on children’s motor performance. In addition, the OTCPDD was developed for documenting the treatment effects of developmental disregard. Further investigation of its clinimetric properties (including the responsiveness and the clinically important difference) is necessary to determine whether it can sufficiently detect changes and whether those changes are clinically relevant.

## Conclusions

In conclusion, the OTCPDD is a reliable and valid observation-based assessment instrument that uses daily bimanual tasks for measuring capacity, performance, and developmental disregard in children with CP. The OTCPDD has several unique features. First, it detects the capacity, performance, and developmental disregard through observation of performing daily bimanual tasks to represent the general affected hand functions in daily routines. Second, the OTCPDD measures a child’s capacity and performance with the same task in both spontaneous use and forced use conditions to reduce the influence of task properties. The OTCPDD can also quantify the severity of developmental disregard and thereby help researchers and clinicians to understand children’s affected hand functions comprehensively and could be used as a potential outcome indicator for treatment effectiveness. Third, the subjective item-matched questionnaire, the DDQ, can provide supplemental information to reflect the parents’ real perceptions of their children’s motor performance in daily life. It is suggested that further studies recruit larger samples of children with CP, examine children at different levels of severity, and examine the clinimetric properties of the OTCPDD.

## Supporting Information

S1 Appendix Items and scoring criteria of the Observatory Test of Capacity, Performance, and Developmental Disregard (OTCPDD). (DOCX)

S2 Appendix Scoring sheet of the Observatory Test of Capacity, Performance, and Developmental Disregard (OTCPDD). (DOCX)

S3 Appendix Questionnaire of Developmental Disregard (QDD). (DOCX)

S1 Dataset (CSV)
