# Supplementary material for: Developing the Observatory Test of Capacity, Performance, and Developmental Disregard (OTCPDD) for Children with Cerebral Palsy
Source: PLoS One. 2016 Mar 24;11(3):e0151798. doi: 10.1371/journal.pone.0151798 (PMC4806991; doi:10.1371/journal.pone.0151798)
Supplement: S1 Appendix — (DOCX) [file pone.0151798.s001.docx]

**S1 Appendix. Items and scoring criteria of the Observatory Test of Capacity, Performance, and Developmental Disregard (OTCPDD)**

| **Items** | |
| --- | --- |
| 1. | Assembling LEGO blocks |
| 2. | Dissembling LEGO blocks |
| 3. | Putting LEGO blocks in a zip-lock bag |
| 4. | Putting toys into a basket |
| 5. | Stringing beads |
| 6. | Drawing on paper |
| 7. | Using an eraser |
| 8. | Using a ruler |
| 9. | Folding a piece of paper |
| 10. | Cutting a piece of paper |
| 11. | Turning a book to a specific page |
| 12. | Opening a bottle |
| 13. | Pouring water from a bottle |
| 14. | Catching and throwing a ball |
| 15. | Putting on a jacket |
| 16. | Zipping up a jacket |
| 17. | Using wet wipes to clean the hands |
| 18. | Unwrapping and eating a piece of candy |
|  | |
| **Scoring criteria** | |
| **Amount of Use (AOU)**  The AOU scores the occurrence of 10 functional motor components (5 basic functions: reach, grasp, hold & carry, release, and stabilize; and 5 advanced functions: adjust, catch/throw, manipulate, press, and pinch). The ten functional motor components are scored on a binary scale (0/1). If the particular motor component is observed at least one time, the item is scored as 1 point, and if the component is not observed, it is scored as 0 points. The maximum score of each item is 10 points. | |
| **Quality of Movement (QOM)**  The QOM is scored on a six-point scale (0-5) to record the general quality of the participants’ movement strategies for each item. The criteria are set in terms of motor coordination, motor accuracy, muscle tone, the role of the affected upper limb, compensatory strategies, and associated movements.   1. A score of 5 points indicates that the performance is equal to that of typically developing children, which means that no abnormal pattern is observed and that both upper limbs play complementary roles of working hands for the intended purposes in an accurate and coordinated fashion. 2. A score of 4 points indicates that both upper limbs play the roles of working hands for the intended purposes, but the movement is slightly slow and uncoordinated, possibly with slightly abnormal patterns. 3. A score of 3 points indicates that the affected upper limb can play the role of assisting hand during the whole process or that the performance is quite slow due to the influence of abnormal muscle tone or patterns. 4. A score of 2 points means that the participant finishes the task with compensatory strategies, including using other body parts or external support to assist the affected upper limb, or that the affected upper limb can only partially play the role of assisting hand. 5. A score of 1 point means that the participant tries to use the affected upper limb but fails to achieve functional use; a score of 0 points indicates that no movements or only associated movements are observed. | |
