# Supplementary material for: Developing the Observatory Test of Capacity, Performance, and Developmental Disregard (OTCPDD) for Children with Cerebral Palsy
Source: PLoS One. 2016 Mar 24;11(3):e0151798. doi: 10.1371/journal.pone.0151798 (PMC4806991; doi:10.1371/journal.pone.0151798)
Supplement: S2 Appendix — (DOCX) [file pone.0151798.s002.docx]

**S2 Appendix. Scoring sheet of the Observatory Test of Capacity, Performance, and Developmental Disregard (OTCPDD)**

| Task/Scores | Amount of Use | | | | | | | | | | Quality of Movement |
| --- | --- | --- | --- | --- | --- | --- | --- | --- | --- | --- | --- |
|  | Reaches | Grasps | Carries/ Holds | Releases | Stabilizes | Adjustments | Catches & Throws | Manipulates | Presses | Pinches |  |
| 1. Assembling LEGO blocks |  |  |  |  |  |  |  |  |  |  |  |
| 1. Dissembling LEGO blocks |  |  |  |  |  |  |  |  |  |  |  |
| 1. Putting LEGO blocks in a zip-lock bag |  |  |  |  |  |  |  |  |  |  |  |
| 1. Putting toys into a basket |  |  |  |  |  |  |  |  |  |  |  |
| 1. Stringing beads |  |  |  |  |  |  |  |  |  |  |  |
| 1. Drawing on paper |  |  |  |  |  |  |  |  |  |  |  |
| 1. Using an eraser |  |  |  |  |  |  |  |  |  |  |  |
| 1. Using a ruler |  |  |  |  |  |  |  |  |  |  |  |
| 1. Folding a piece of paper |  |  |  |  |  |  |  |  |  |  |  |
| 1. Cutting a piece of paper |  |  |  |  |  |  |  |  |  |  |  |
| 1. Turning a book to a specific page |  |  |  |  |  |  |  |  |  |  |  |
| 1. Opening a bottle |  |  |  |  |  |  |  |  |  |  |  |
| 1. Pouring water from a bottle |  |  |  |  |  |  |  |  |  |  |  |
| 1. Catching and throwing a ball |  |  |  |  |  |  |  |  |  |  |  |
| 1. Putting on a jacket |  |  |  |  |  |  |  |  |  |  |  |
| 1. Zipping up a jacket |  |  |  |  |  |  |  |  |  |  |  |
| 1. Using wet wipes to clean the hands |  |  |  |  |  |  |  |  |  |  |  |
| 1. Unwrapping and eating a piece of candy |  |  |  |  |  |  |  |  |  |  |  |
