# Supplementary material for: Developing the Observatory Test of Capacity, Performance, and Developmental Disregard (OTCPDD) for Children with Cerebral Palsy
Source: PLoS One. 2016 Mar 24;11(3):e0151798. doi: 10.1371/journal.pone.0151798 (PMC4806991; doi:10.1371/journal.pone.0151798)
Supplement: S3 Appendix — (DOCX) [file pone.0151798.s003.docx]

**S3 Appendix. Questionnaire of Developmental Disregard (QDD)**

| **Items** | | **How Often** | **How Well** |
| --- | --- | --- | --- |
| 1. | Assembling LEGO blocks |  |  |
| 2. | Dissembling LEGO blocks |  |  |
| 3. | Putting LEGO blocks in a zip-lock bag |  |  |
| 4. | Putting toys into a basket |  |  |
| 5. | Stringing beads |  |  |
| 6. | Drawing on paper |  |  |
| 7. | Using an eraser |  |  |
| 8. | Using a ruler |  |  |
| 9. | Folding a piece of paper |  |  |
| 10. | Cutting a piece of paper |  |  |
| 11. | Turning a book to a specific page |  |  |
| 12. | Opening a bottle |  |  |
| 13. | Pouring water from a bottle |  |  |
| 14. | Catching and throwing a ball |  |  |
| 15. | Putting on a jacket |  |  |
| 16. | Zipping up a jacket |  |  |
| 17. | Using wet wipes to clean the hands |  |  |
| 18. | Unwrapping and eating a piece of candy |  |  |
|  | | | |
| **Scoring criteria** | | | |
| **How-Often Scale**   1. Never used weaker arm when the task was attempted.   0.5-   1. Seldom used weaker arm when attempting the task (1-2 times out of 10)   1.5-   1. Used weaker arm in ~ 25% of the attempts (3-4 times out of 10)   2.5-   1. Used weaker arm in half of the attempts (5-6 times out of 10)   3.5   1. Used weaker arm in most of the attempts (7-8 times out of 10)   4.5-   1. Used weaker arm in almost all of the attempts (9-10 times out of 10) | | | |
| **How-Well Scale**   1. Weaker arm not used at all for the activity (no use)   0.5-   1. Weaker arm moved for the activity, but was not helpful (very poor)   1.5-   1. Weaker arm of some use in the activity, but needed help from the stronger arm or moved very slowly or with difficulty (poor)   2.5-   1. Weaker arm used for the activity, but movements were slow or were made with only some effort (fair)   3.5   1. Movements made by weaker arm for the activity almost typical for age, but not quite as fast or accurate (almost normal)   4.5-   1. Ability to use the weaker arm for that activity typical for age (normal) | | | |
